# Navigating Central Oxytocin Transport: Known Realms and Uncharted Territories

**DOI:** 10.1177/10738584241268754

**Published:** 2024-08-07

**Authors:** Deniz Parmaksiz, Yongsoo Kim

**Affiliations:** 1 Department of Neural and Behavioral Sciences, College of Medicine, The Pennsylvania State University, Hershey, PA, USA; 2 Center for Neural Engineering, The Pennsylvania State University, University Park, PA, USA

**Keywords:** oxytocin, transport mechanism, volume transmission, axonal release, somatodendritic release, cerebrospinal fluid, neurovasculature, perivascular space, blood-brain barrier, circumventricular organs, neurohypophyseal system, hypothalamus, neuroendocrinology

## Abstract

Complex mechanisms govern the transport and action of oxytocin (Oxt), a neuropeptide and hormone that mediates diverse physiologic processes. While Oxt exerts site-specific and rapid effects in the brain via axonal and somatodendritic release, volume transmission via CSF and the neurovascular interface can act as an additional mechanism to distribute Oxt signals across distant brain regions on a slower timescale. This review focuses on modes of Oxt transport and action in the CNS, with particular emphasis on the roles of perivascular spaces, the blood-brain barrier (BBB), and circumventricular organs in coordinating the triadic interaction among circulating blood, CSF, and parenchyma. Perivascular spaces, critical conduits for CSF flow, play a pivotal role in Oxt diffusion and distribution within the CNS and reciprocally undergo Oxt-mediated structural and functional reconstruction. While the BBB modulates the movement of Oxt between systemic and cerebral circulation in a majority of brain regions, circumventricular organs without a functional BBB can allow for diffusion, monitoring, and feedback regulation of bloodborne peripheral signals such as Oxt. Recognition of these additional transport mechanisms provides enhanced insight into the systemic propagation and regulation of Oxt activity.

## Introduction

**Figure 1.**
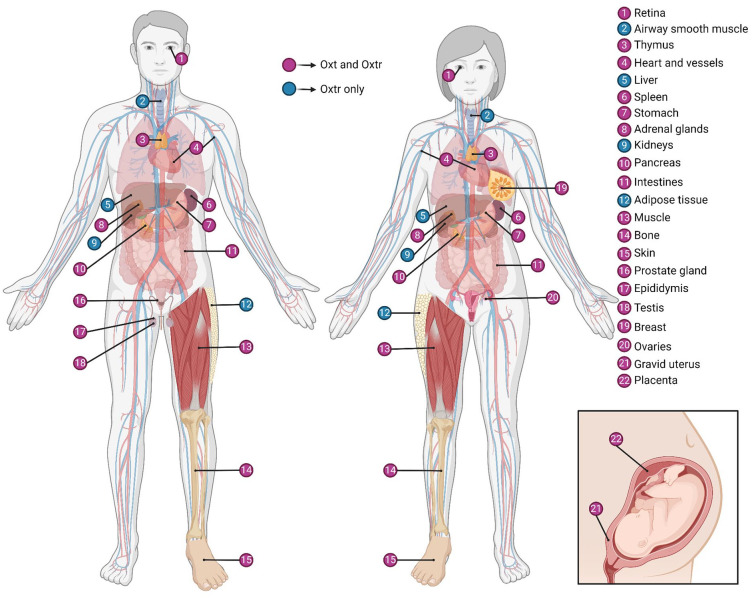
Oxytocin and oxytocin receptor expression in the body. For information on expression in each peripheral site, refer to Supplementary Table S1. Oxt = oxytocin; Oxtr = oxytocin receptor. Created with BioRender.com.

Oxytocin (Oxt), a highly conserved neuropeptide and hormone, is widely recognized for its pivotal roles in mammalian reproduction and prosocial behaviors (Box 1). Within the CNS, Oxt is predominantly synthesized in the hypothalamus, notably the supraoptic nucleus (SON) and paraventricular (PVH) regions, alongside several accessory nuclei (Grinevich and others 2016; Jurek and Neumann 2018; Son and others 2022). Hypothalamic Oxt is supplemented by Oxt locally produced in various peripheral sites across the reproductive, cardiovascular, gastrointestinal, immune, and musculoskeletal systems of both sexes (Assinder 2022; Figure 1).

**Box 1.** Oxytocin.

In 1906, Sir Henry Dale noted that extracts from the human posterior pituitary caused uterine contractions, leading to the discovery of oxytocin (Oxt), a highly conserved nonapeptide hormone derived from the larger precursor protein neurophysin (Brownstein and others 1980; Dale 1906). Often referred to as the “love hormone” or “bonding hormone” due to its prosocial effects, Oxt’s influence extends across a spectrum of behavioral, cognitive, and physiologic processes, encompassing parturition, lactation, pair bonding, parental behaviors, sociability, anxiety regulation, appetite control, and body fluid homeostasis (Gimpl and Fahrenholz 2001; Jurek and Neumann 2018; Quintana and Guastella 2020; Son and others 2022). These effects are mediated by the Oxt receptor, a G protein–coupled receptor ubiquitously expressed throughout the brain and body (Jurek and Neumann 2018). Disruptions in Oxt signaling have been linked to several conditions, ranging from autism spectrum disorders to preeclampsia, prompting research into the therapeutic potential of exogenous Oxt treatment (Ghazy and others 2023; D. A. Martins and others 2020; Martins and others 2022).

**Figure 2.**
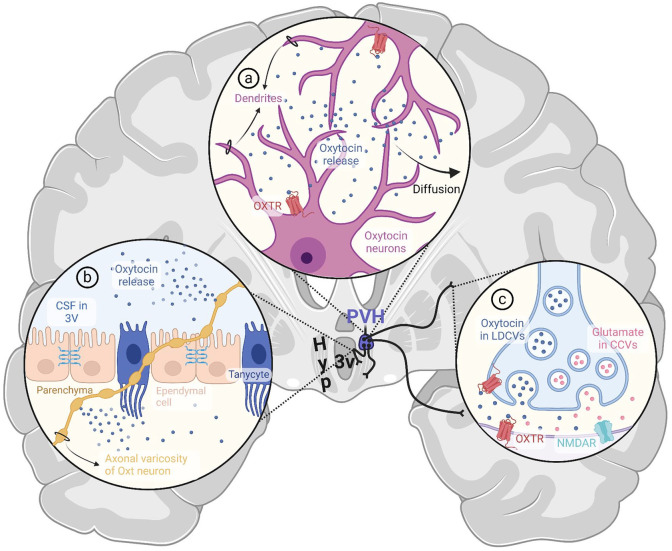
Established modes of central oxytocin transport and action. (a) Somatodendritic release. Oxytocin released from dendrites of magnocellular and parvocellular neurons of hypothalamic nuclei engages in autocrine and paracrine signaling by activating oxytocin receptors on the neuron of origin and its neighbors. The remaining oxytocin diffuses from the parenchyma into the CSF. (b) En passant release. Oxytocin can be released extrasynaptically from undilated axons and axonal varicosities. Several axon collaterals of oxytocinergic neurons are strategically positioned to facilitate en passant release directly into the third ventricle or subarachnoid space, thereby contributing to volume transmission of oxytocin in CSF. (c) Synaptic release. Axons of parvocellular oxytocin neurons make synaptic contacts in various brain regions, including the isocortex, cortical subplate, hippocampal formation, pallidum, striatum, midbrain, brainstem, and spinal cord. In some species, cortical and subcortical projections of magnocellular neurons have also been reported. Upon release from synaptic terminals, oxytocin can bind to pre- and postsynaptic oxytocin receptors. Oxytocin is thought to mediate NMDAR-dependent plasticity via co-release with glutamate at specific synapses within the frontal cortex. Spillover from the synaptic cleft can also contribute to volume transmission via CSF. 3V = third ventricle; CCV = clear core vesicle; Hyp = hypothalamus; LDCV = large dense core vesicle; NMDAR = N-methyl-D-aspartate receptor; Oxt = oxytocin; OXTR = oxytocin receptor; PVH = paraventricular nucleus of the hypothalamus. Created with BioRender.com.

In the CNS, Oxt acts as a neuromodulator through two complementary mechanisms. Somatodendritic Oxt release mediates localized effects in the hypothalamus and neighboring brain areas, fine-tuning the sensitivity of the Oxt system, while axonal transmission extends Oxt signaling to remote brain regions (Grinevich and others 2016; Figure 2). Furthermore, synaptically and dendritically released Oxt can diffuse into the CSF, constituting a third mode of Oxt transport known as volume transmission (Box 2), through which Oxt functions as a hormone, eliciting widespread effects on a slower timescale (Veening and others 2010). Notably, as CSF traverses perivascular spaces, Oxt, known for its cardiovascular effects (Gutkowska and Jankowski 2012), intimately and reciprocally interacts with cerebral circulation (Alonso and others 2005; Miyata and Hatton 2002). Despite extensive research on Oxt transport in CSF, the neurovascular interface’s role in volume transmission and overall central Oxt function has been largely neglected. Additionally, significant efforts are needed to understand the interplay between central and peripheral Oxt systems and the blood-brain barrier’s (BBB) permeability to Oxt, particularly regarding exogenous Oxt treatments for neuropsychiatric disorders (D. A. Martins and others 2020; Valstad and others 2017; Yamamoto and others 2019). However, studies examining potential transport mechanisms often overlook circumventricular organs (CVOs), specialized structures devoid of a functional BBB, which are anatomically connected with oxytocinergic nuclei and share significant functional overlap with the Oxt system (Jeong and others 2021; Sisó and others 2010). This review explores the mechanisms governing Oxt transport and activity in the CNS, with a focus on the crucial roles of the neurovascular unit, emphasizing perivascular spaces, the BBB, and CVOs.

**Box 2.** Volume Transmission.

*Volume transmission* refers to a mode of communication within the brain that involves the diffusion of signaling molecules through the extracellular fluid and CSF rather than traditional point-to-point synaptic transmission (Agnati and others 1986; Agnati and others 2010). Volume transmission encompasses various release mechanisms beyond direct delivery into the CSF, with contributions from somatodendritic release, en passant (or extrasynaptic) release from axonal collaterals, and spillover from synaptic transmission. Diffusion of neurotransmitters and peptides is mediated by flow along spaces surrounding blood vessels and myelinated fiber bundles, as influenced by many factors, including concentration and pressure gradients (Iliff and others 2012; Iliff and others 2013). Key diffusion parameters include 1) volume fraction of extracellular fluid and CSF as compared with total brain volume; 2) clearance rate of neurotransmitters; and 3) tortuosity, which describes increases in diffusional path length due to extracellular matrix components and glial processes (Agnati and others 1986; Agnati and others 2010). Unlike synaptic transmission, which is rapid, precise, and localized, volume transmission allows for more widespread and flexible modulation of neural circuits across an extended timescale (Zoli and others 1999). Because of its brainwide effects, volume transmission is thought to play a crucial role in modulating mood and arousal.

## Axonal and Somatodendritic Transmission

Axons of Oxt magnocellular neurons (MCNs) residing in the PVH extend ventrally and intersect with supraoptic projections, establishing the hypothalamoposthypophyseal tract (HPH; Carey 1959; Currie and others 1960). These fibers terminate as Herring bodies in the posterior pituitary, where “burst firing” or synchronized high-frequency Oxt release into plasma elicits well-established physiologic responses, such as milk ejection and uterine contraction (Summerlee 1981; Wakerley and Lincoln 1973). Oxt, implicated in various bodily functions in both sexes, is continuously secreted into the bloodstream to sustain basal concentrations of 1 to 3 pg/mL by Oxt MCNs, firing at a rate of ~1 to 5 spikes per second (Brown 2016).

The traditional view held that MCN axons exclusively target the posterior pituitary and release Oxt somatodendritically (Figure 2a), while parvocellular neurons (PCNs) send out central projections to regulate Oxt’s behavioral effects (Swanson and Sawchenko 1980, 1983). This view has been challenged by the detection of MCN axon collaterals in the rat brain that extend to cortical areas (e.g., piriform and auditory cortices) and subcortical regions such as the central amygdala, caudate putamen, and nucleus accumbens (Knobloch and others 2012; Zhang and others 2021). However, recent investigations in mice report that PVH MCNs projecting to the pituitary do not extend axonal arbors to extrahypothalamic regions (Li and others 2024), possibly highlighting interspecies differences in morphologic patterns and connectivity of Oxt neurons. Conversely, projections of PCNs have been reported in the isocortex, cortical subplate, hippocampal formation, pallidum, striatum, midbrain, brainstem, and spinal cord (Althammer and Grinevich 2017; Hosoya and others 1991; Li and others 2024; Pyner and Coote 2000; Shafton and others 1998; Swanson and Sawchenko 1980, 1983; Xiao and others 2017), where Oxt action can regulate nociception (Eliava and others 2016; Iwasaki and others 2023), satiety (Blevins and others 2004; Uchoa and others 2009), and cardiovascular and autonomic processes (Mack and others 2002; Roy and others 2018; Xu and others 2012). PCNs are thought to not only receive and integrate sensory information but also modulate MCN activity somatodendritically (Eliava and others 2016), and they have been implicated in social behaviors (Loth and others 2014; Tang and others 2020; Wei and others 2021) and dysfunction thereof in animal models of autism spectrum disorder (Lewis and others 2020; Tsurutani and others 2023). Furthermore, continued research into morphoelectric characteristics of Oxt neuron populations and application of advanced methods, such as single-cell and nucleus RNA sequencing, have begun to shed light to the structural, functional, and genetic diversity of Oxt neurons, identifying several types of Oxt neurons beyond the canonical dichotomous categorization (Althammer and Grinevich 2017; Chen and others 2022; Xu and others 2020).

It remains uncertain whether MCNs make synaptic contacts outside the posterior pituitary (Li and others 2024), despite reports of such contacts in the central amygdala (Knobloch and others 2012; Morris and Pow 1991), which may serve a modulatory function via co-release of glutamate with Oxt (Figure 2c), as supported by expression of the glutamate transporter VGLUT2 (Hrabovszky and Liposits 2008). The remainder of Oxt from MCN axons is released extrasynaptically, acting on glutamatergic and GABAergic synapses that show abundant Oxt receptor (Oxtr) expression in pre- and postsynaptic terminals (Mitre and others 2016). This constitutes en passant release (Figure 2b), or exocytosis from varicosities and undilated axons, which may not only mediate social fear inhibition in the lateral septum but also contribute to volume transmission of Oxt via CSF (Chini and others 2017; Li and others 2024; Menon and others 2018), as elaborated in the next section.

Besides their known role in receiving and propagating synaptic inputs, dendrites are capable of synthesizing and releasing neurotransmitters (Gao 1998), which then exert autocrine and paracrine effects by acting on pre- and postsynaptic terminals (Ludwig and Leng 2006). This mechanism, known as *somatodendritic release*, accounts for a majority of Oxt output within the CNS (Figure 2a) and has been extensively studied and reviewed in the context of Oxt signaling (Brown and others 2020; Ludwig and Leng 2006; Pow and Morris 1989).

Unlike neurotransmitters, neuropeptides such as Oxt are stored in large dense-core vesicles (LDCVs) that do not require vesicular transport proteins (Li and Kim 2008) and can be released from all neuronal compartments, including dendrites (Morris and Pow 1991). Somatodendritic release of Oxt LDCVs is regulated by multiple mechanisms. As in synaptic release, action potential–mediated calcium influx through voltage-gated channels prompts exocytosis of LDCVs (Ludwig and Leng 2006). The activation of NMDARs (N-methyl-D-aspartate receptors), which contribute to calcium influx, is required for somatodendritic transmission at basal firing rates and enhances activity-driven release (Pitra and others 2019), presumably by increasing LDCV trafficking velocities (Kirchner and others 2023). Additionally, Oxt MCNs express Oxtrs (Freund-Mercier and others 1994), which, upon activation (e.g., in lactating females), prompt further Oxt release by de-sequestering intracellular calcium stores via dual actions of CD38-dependent second messengers cADPR and NAADP (Lopatina and others 2010; Salmina and others 2010) and the IP3 pathway (Dayanithi and others 2000). This priming process involves the transport of LDCVs to the readily releasable pool, as well as direct release from the reserve pool (Sedej and others 2009; Tobin and others 2004), accompanied by heightened density of N-type calcium channels, which are functionally implicated in dendritic exocytosis (Tobin and others 2011). Cytoskeletal network restructuring, particularly fluctuations in F-actin polymerization (Tobin and Ludwig 2007) and microtubule density (Hicks and others 2020), further modulate somatodendritic release under various physiologic conditions, such as salt loading.

Oxt action on its cell of origin and neighboring cells can have diverse effects. Under basal conditions, Oxtr binding suppresses excitatory postsynaptic currents and promotes endocannabinoid release, leading to retrograde inhibition of afferent terminals and reduced excitatory tone through negative feedback (Hirasawa and others 2001; Hirasawa and others 2004). However, under osmotic stimulation, endocannabinoid action can switch from inhibition to excitation via TRPV-1 channel activation (Sharif Naeini and others 2006). Likewise, GABAergic transmission becomes excitatory during pregnancy, as accompanied by augmented glutamatergic inputs onto MCNs (Lee and others 2015). These electrochemical adjustments and priming mechanisms can create a positive feedback loop for somatodendritic Oxt release under physiologic conditions such as pregnancy, lactation, and osmotic stimulation, which also trigger enhanced Oxt release into plasma from synaptic terminals in the posterior pituitary (Althammer and others 2021; Landgraf and others 1988). Yet, the two modes of release are not always synchronized (Neumann and others 2008; Valstad and others 2017). For example, axonal secretion into the posterior pituitary lags behind the somatodendritic response to suckling in lactating rats (Moos and others 1989), whereas upsurge in plasma Oxt levels precedes dendritic release during osmotic stimulation (Ludwig and others 1994). Furthermore, electrical decoupling between dendrites and other cellular compartments through regulatory mechanisms (i.e., NMDAR gating of dendritic release probability; Pitra and others 2019) and modulation of microgeometrical dendritic factors such as stem diameter, varicosities, and glial sheath wrapping size (Korogod and others 2023) allows liberation of dendritic intracellular calcium stores independent of action potentials. Additionally, α-melanocyte–stimulating hormone, originating from proopiomelanocortin neurons of the arcuate nucleus, promotes somatodendritic Oxt release under basal conditions by elevating intracellular calcium levels while lowering the probability of action potential generation and thus peripheral secretion (Sabatier and Leng 2006; Sabatier and others 2003).

The fine-tuned process of somatodendritic Oxt release in turn regulates the activity of other neurons, especially PCNs. Corticotropin-releasing hormone–secreting PCNs in the PVN are subject to attenuation of afferent glutamatergic inputs upon Oxt binding, potentially suppressing the hypothalamic-pituitary-adrenal axis and mitigating the stress response (Dabrowska and others 2011). Somatodendritic Oxt can also reach brain areas neighboring the PVN and SON that express Oxtrs such as the ventromedial hypothalamus and the medial nucleus of the amygdala, where it may mediate effects on satiety and social recognition, respectively (Klockars and others 2017; Manning and others 2012; Patisaul and others 2003; Takayanagi and others 2017).

Although the influence of somatodendritic Oxt in such regions adjacent to SON and PVN is well established, its ability to reach distant brain areas is still debated, as detailed in the next section.

## Volume Transmission via CSF

Volume transmission of neuropeptides such as Oxt in CSF has long been recognized as a counterpart to axonal projections, which may exert lasting changes to neural function and behavior by operating across a broader spatial and temporal scale (Box 2; Agnati and others 1986; Agnati and others 2010). Oxt has been detected in the CSF of many species, including humans, primates, and rodents (Amico and others 1983; Artman and others 1982; Devarajan and Rusak 2004), with microdialysis studies confirming its effective diffusion into CSF and brain regions beyond the PVH and SON (Ebner and others 2000; Engelmann and others 2006). Yet, some argue that basal CSF Oxt levels fall significantly below the threshold needed to activate Oxtrs across the brain, with constraints from peptidases and tissue-specific diffusion barriers confining its effects locally (Chini and others 2017). In vivo responses are typically observed within the range of 1 to 100nM Oxt, consistent with Oxtr ligand-binding assays (Elands and others 1988), but significantly higher than picomolar CSF Oxt concentrations (Monte and others 2014; Striepens and others 2013). In vivo electrophysiology experiments typically report effects at 1 to 5μM concentrations due to limited peptide penetration, which may yield conflicting results, as distinct Oxtr signaling cascades can be activated at different Oxt concentrations (Busnelli and others 2012; Chini and others 2017). However, CSF measurements might be underestimated, based on the low recovery rate (~10%) of microdialysis probes (Landgraf and Ludwig 1991). Recent technological advancements, such as the development of fluorescent sensor MTRIAOT (Ino and others 2022) and the genetically encoded sensor GRABOT (Qian and others 2023), have enabled the measurement of extracellular Oxt and the detection of Oxt release with high spatiotemporal resolution. These sensors offer significant potential for elucidating CSF Oxt concentrations and determining whether CSF Oxt reaches brain areas such as the somatosensory or primary motor cortices, which lack oxytocinergic projections. Yet, their current sensitivity is in the nanomolar range, whereas basal CSF Oxt concentrations are believed to be in the picomolar range. Consequently, while these sensors are likely to perform well in brain regions with consistently high Oxt concentrations, such as the PVH and SON, and show promise in detecting activity-dependent increases in Oxt release, enhancing their sensitivity is crucial to resolving the CSF Oxt debate under basal conditions. Another alternative was pioneered by Nakamura and colleagues (2022): click chemistry, whereby Oxt tagged with a small alkyne molecule is applied to acute brain slices, fixed, and conjugated with azide-tagged fluorophores. These authors identified high-affinity binding sites in the hippocampus, which respond to low concentrations of Oxt presumed to reach the target receptors through volume transmission, as supported by fast turnover of Oxt binding and lack of uptake into cells. As noted by the authors, click chemistry could also be used in vivo to explore Oxt delivery via CSF and shed light on spatiotemporal dynamics of extracellular Oxt in different sites of action.

As alluded to in the hippocampus example, variability in receptor expression, efficacy, and binding sites across brain regions and physiologic states can influence the sensitivity of different areas to identical Oxt concentrations (Jurek and Neumann 2018; Meddle and others 2007). Oxt binding induces diverse conformational changes in Oxtrs, activating various downstream effectors (Reversi and others 2005). While Gi and β-arrestins require higher concentrations for activation, the Gq pathway can be turned on by lower levels of Oxt, particularly by homodimeric Oxtrs (~1000-fold lower), as compared with monovalent forms (Busnelli and others 2012; Busnelli and others 2016). Gq signaling is the primary driver of Oxt action, inducing calcium influx and downstream proliferative effects (Wang and Hatton 2007), although elevated Oxt levels or enhanced Gi expression can modify this response (Busnelli and others 2012). For instance, atosiban, once viewed as an Oxtr antagonist, is now recognized as a biased agonist that selectively engages the Gi pathway upon Oxtr binding to increase cytokine synthesis and inhibit cell growth (Kim and others 2016; Reversi and others 2005). Furthermore, owing to structural homology, high Oxt concentrations can activate arginine vasopressin (Avp) receptors, especially vasopressin receptor 1A (V1aR), and induce a response similar to Gi signaling (Chini and others 2017; Manning and others 2012; Neumann and Landgraf 2012). Hence, under basal conditions, low CSF Oxt levels selectively recruit the Oxtr-Gq pathway, while activity-dependent increases in Oxt concentrations may engage Oxtr-Gi and V1aR in select brain areas to modulate region-specific responses.

The efficacy of volume transmission is expected to be further influenced by various regulatory mechanisms, such as steroid hormone action (Gimpl and Fahrenholz 2001), that govern local receptor sensitivity and recycling dynamics (Conti and others 2009). Such an influence of steroids is evident in the ventromedial hypothalamus and preoptic area, where receptor sensitivity fluctuates throughout the estrous cycle, affecting the responsiveness of these regions to basal Oxt levels (Bale and others 1995; Caldwell and others 1994; Jirikowski and others 2018). On top of these considerations, significant variations (up to fivefold) in diurnal CSF Oxt levels complicate the definition of “basal” levels (Artman and others 1982; Devarajan and Rusak 2004). Even if basal concentrations are insufficient to elicit a brainwide response, CSF could propagate activity-dependent increases in Oxt signaling, turning local signals into global responses during states of heightened Oxt release, as evidenced by elevated CSF Oxt levels during osmotic stimulation and nursing (Kendrick and others 1987; Morris and others 1984).

Volume transmission of Oxt is supported by the consistently reported mismatch between Oxt projections and Oxtr expression (Herkenham 1987; Manjila and others 2022; Son and others 2022). For instance, the olfactory bulb, crucial for social bonding and parental behavior in rodents (Meddle and others 2000), exhibits abundant Oxtr expression despite lacking direct projections from hypothalamic Oxt neurons (Son and others 2022), lending support to the functional significance of volume transmission of Oxt via CSF.

In accordance, the SON and PVH, which house most Oxt neurons, are strategically located near the ventral subarachnoid space and third ventricle (3V), respectively, facilitating interaction with CSF (Veening and others 2010). While Oxt PCNs project to ventricles and other ventricular system–related areas, dendrites of MCNs in the PVH extend centromedially, penetrating the ventricular lining either subependymally or running along its surface to reach the supraependymal plexus on the 3V floor (Buijs 1978; Cloft and Mitchell 1994; Ju and others 1992; Krisch 1986; Li and others 2024). Some dendritic processes and axon collaterals reach across to the dorsal 3V (Rhodes and others 1981), contributing to bilateral PVH synchronization in lactating females (Belin and Moos 1986). SON MCNs also project dendritic processes toward the ventral surface and into the subarachnoid space, instrumental to contralateral SON activation during unilateral Oxt neuron stimulation in lactating animals (Neumann and others 1995). Dendritic processes of periventricular nucleus Oxt neurons penetrate the ventricular lumen as well (Jirikowski 2019; Vigh-Teichmann and others 1970). Furthermore, axon terminals from SON and PVH can contact CSF through gaps in the ependymal lining of the infundibular recess, a CSF-filled space connecting the 3V to the pituitary (Jirikowski 2019; Wittkowski 1969).

While the anatomic features detailed here facilitate the direct release of Oxt into CSF, these examples alone are insufficient to supply adequate Oxt for diffusion into distant brain regions. Somatodendritic release, accounting for 95% of central Oxt output (Rossoni and others 2008), is considered the primary source of CSF Oxt (Veening and others 2010; Figure 2a), although there are notable constraints in the time frame and effectiveness of this route. Diffusion of dendritically released Oxt into CSF and brain regions is hindered by factors such as tortuosity (i.e., diffusional path length) of the extracellular space and local variations in aminopeptidase expression. On one hand, only about 10% to 15% of extracellular fluid drains into the ventricular system (Weller and others 2009), and Oxt has a short half-life (<1 minute) in parenchyma (Stark and others 1989). On the other, continuous LDCV exocytosis sustains peptide availability for diffusion (Xia and others 2009), and the fraction reaching CSF can circulate longer due to a protracted half-life (~25 minutes; Mens and others 1983). Furthermore, additional factors, such as glial retraction, can substantially broaden the range of diffusion and increase Oxt release probability (Theodosis 2002). Oxt availability in parenchyma is also significantly influenced by levels of placental leucine aminopeptidase (P-LAP), which degrades Oxt (Matsumoto and others 2001). Despite being present in all Oxtr-expressing brain regions, P-LAP concentrations do not correlate with Oxtr density (Grinevich and others 2016). Although P-LAP decreases Oxt availability at excess, moderate levels can coordinate lactation-induced burst firing and enhance tissue sensitivity by countering Oxtr desensitization (Beyer and others 2010; Tobin and others 2014).

Others in the field postulate that axonal, specifically en passant, release (Figure 2b and 2c) contributes significantly to volume transmission (Chini and others 2017), challenging the predominant view of somatodendritic release in the SON and PVH as the main source of CSF Oxt. While en passant release may explain some Oxt actions previously attributed to somatodendritic release (Li and others 2024), such as satiety signaling through transsynaptic co-release of glutamate in the ventromedial hypothalamus (Griffin and others 2010), sparse collaterals of Oxt axons are unlikely to maintain basal CSF Oxt levels alone. It is likely that the two modalities work together such that somatodendritic transmission establishes a baseline and fine-tunes excitation, while axonal release provides precise control over timing and location, efficiently targeting Oxtrs in distant brain regions.

## Transmission via Perivascular Spaces, Circulation, and Circumventricular Organs

The peripheral role of Oxt as a hormone is well established, as is the close interaction between the ventricular system and cerebral vessels. However, the role of circulation in central Oxt transport, whether it be direct through blood flow or indirect via CSF movement through perivascular spaces, is often overlooked.

While CSF production was historically thought to be limited to the choroid plexus and ependymal cells of the ventricular system, with negligible contributions from parenchyma (McComb 1983), recent evidence suggests that the walls of CNS capillaries are responsible for a bulk of interstitial fluid and CSF formation and reabsorption (Hladky and Barrand 2016; Oresković and Klarica 2010). In a similar vein, although CSF absorption into circulation is traditionally thought to occur across arachnoid villi (Davson and others 1973) and cervical lymphatics (McComb 1983), studies with radioiodinated albumin have shown notable drainage through cerebral perivascular spaces and olfactory lobe subarachnoid spaces (Bradbury and others 1981), highlighting plasma and CSF as interconnected modes of molecule transport within the CNS.

### Perivascular Spaces

**Figure 3.**
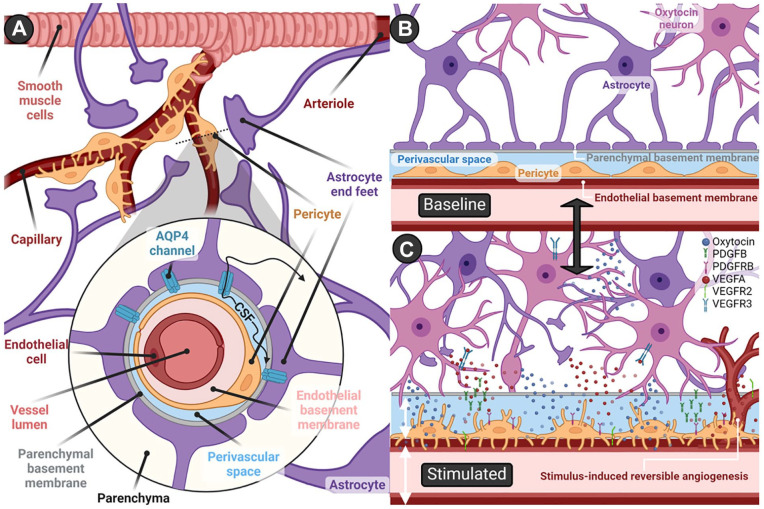
Perivascular spaces and stimulus-induced neurovascular remodeling. (a) Schematic representation of the perivascular space. CSF flows within perivascular spaces that surround cerebrovasculature before drainage into the cervical lymphatic system. At the level of arteries, the inner border of perivascular spaces is demarcated by the pial sheath, which disappears and yields to the endothelial basement membrane at the capillary and venous levels. The outer boundary is formed by astrocyte end feet, which constitute the glia limitans of the blood-brain barrier. Aquaporin 4 channels mediate CSF movement between perivascular spaces and parenchyma, with direction and velocity of flow determined by various factors, including arterial pulsations, pressure gradients, respiration, and neural activity. (b) Oxytocin neurons and the perivascular space at baseline activity levels. (c) Stimulus-induced neurovascular remodeling and angiogenesis. Reversible changes to the neurovascular unit have been reported in the hypothalamus and posterior pituitary after activity-dependent increases (e.g., lactation, salt loading) in oxytocin signaling—namely, retraction of astrocyte end feet and concurrent extension of pericyte processes. Subsequent to this surface expansion, dendrites and axons of oxytocin neurons come in close contact with the perivascular space and endothelial basement membrane, facilitating diffusion of oxytocin into the CSF and potentially enabling penetration into vessels. The reversible increases in capillary density and neurovascular remodeling are orchestrated by the upregulated expression of angiogenic factors: VEGFA in astrocytes; VEGFR2, PDGFB, and PDGFRB in endothelial cells; PDGFB and VEGFR3 in oxytocin neurons; and PDGFRB in pericytes. AQP4 = aquaporin 4; PDGFB = platelet-derived growth factor β; PDGFRB = platelet-derived growth factor receptor; VEGFA = vascular endothelial growth factor α; VEGFR2 = vascular endothelial growth factor receptor 2; VEGFR3 = vascular endothelial growth factor receptor 3. Created with BioRender.com.

Perivascular space, also known as *Virchow-Robin space* (Weed 1923), describes the area between astrocyte end feet that make up glia limitans and either the pial sheath (in arteries) or endothelial basement membrane (in capillaries and veins; Ineichen and others 2022; Figure 3). There has been considerable confusion about the terminology, structure, and function of perivascular spaces, with challenges in their distinction from other spaces made up of layers of basement membranes enclosed by smooth muscle cells around arteries, as well as questions regarding the plausibility of circulation driven by steady pressure in either space (Faghih and Sharp 2018). Here, we refer to perivascular space to describe extensions of the subarachnoid spaces surrounding cerebrovasculature, which follow the course of penetrating arteries down to the level of capillaries (Hadaczek and others 2006), making up the so-called glymphatic system (Jessen and others 2015), implicated in the flow of CSF from the subarachnoid space into parenchyma (Rennels and others 1985), as well as clearance of substances such as amyloid β from interstitial fluid into CSF (Hawkes and others 2014; Weller and others 2009). This conceptual framework suggests that CSF permeates into perivascular spaces around penetrating arteries, moves into parenchyma via aquaporin 4 channels (Iliff and others 2012; Figure 3a), and flows out through perivascular spaces surrounding deep veins to be drained into the cervical lymphatic system (Bacyinski and others 2017; Jessen and others 2015). The precise mechanisms underlying perivascular CSF flow remain a subject of ongoing debate: arterial pulsations were initially thought to be a main driver (Hadaczek and others 2006; Iliff and others 2013); however, arteriolar wall movements were found to propel oscillatory flow but not directional pumping (Kedarasetti, Drew, and others 2020), with suggestions that directional CSF flow may instead be explained by pressure differences, respiration, peristaltic motion in perivascular networks (Gjerde and others 2023), or “valves” formed by astrocyte end feet (Gan and others 2023), while functional hyperemia following neural activity may serve to promote CSF transport and metabolite clearance (Kedarasetti, Turner, and others 2020; Kim and others 2023).

Perivascular clusters of Oxt neurons that extend projections into the vascular endothelium (e.g., circular nucleus; Møller and others 2018; Rovsing and others 2013) can be found in between the PVH and SON as well as in other accessory areas, mainly in preoptic and tuberal regions in rodents (Palkovits and others 1974; Sofroniew and Glasmann 1981), primates (Ichimiya and others 1988), and humans (Krolewski and others 2010). While some of their projections are thought to join axons from the canonical oxytocinergic nuclei and terminate at the posterior pituitary, others may penetrate perivascular spaces, releasing Oxt directly into cerebrovasculature (Jirikowski 2019; Møller and others 2018) or CSF (Morris and others 1998; Figure 3b and 3c). This is exemplified by the highly efficient delivery of CSF messages to ventromedial medullary neurons involved in synchronization of bilateral Oxt-secreting hypothalamic nuclei, thought to be facilitated by the vascular pump (Moos and others 2004). Additionally, increased numbers of Oxt neurons surrounding vasculature have been noted in various hypothalamic nuclei of parturient rats (Jirikowski and others 1988), hinting at a role for perivascular Oxt release in child delivery. Studies utilizing electron microscopical immunocytochemistry and morphometry (Blanco and others 1991) showed that in late pregnancy and after 2 days of lactation, numerous Oxt neurons can be found in direct contact with the outer basement membrane of vessels, at a much lower distance than that seen in ovariectomized controls and even after 9 days of lactation. These transitory changes may be driven by steroid-induced glial retraction (Hatton and others 1984) and may serve to enhance activity-dependent somatodendritic Oxt release (Kamei 1986), which could then diffuse into CSF and potentially supplement plasma Oxt levels (Wagner and others 1974). Through simultaneous release into circulation and CSF, perivascular Oxt neurons could serve to synchronize systemic and central Oxt actions under special physiologic circumstances, such as parturition and lactation.

Additionally, perivascular Oxt release may serve to modulate cerebral perfusion pressure and cerebrovascular tone through endothelial cell (EC) Oxtr signaling (Schini and others 1990; Thibonnier and others 1999), inducing endothelial nitric oxide (NO) synthase production (Cattaneo and others 2009), and calcium-dependent vasodilation and trophic responses (Thibonnier and others 1999). This could have implications for cerebral circulation of not only blood but also CSF: functional hyperemia—that is, elevated local blood flow triggered by neuronal (specifically sensorimotor) activity—is thought to increase CSF circulation and metabolite exchange (Kedarasetti, Turner, and others 2020; Kim and others 2023), and several types of sensory stimulation are known to trigger Oxt release (e.g., suckling, scent of offspring, physical touch; Uvnäs Moberg and others 2022). Oxt-positive feedback on its own release may hence be reinforced by CSF-perivascular mechanisms, whereby somatosensory activity leads to Oxt release and the ensuing functional hyperemia and Oxt-induced vasodilation promote further Oxt neuron activity and neuropeptide diffusion in CSF.

Evidence backing this idea is present in studies of hyperosmotic stimulation, a known trigger of Oxt signaling (Balment and others 1980; Balment and others 1986; Ueno and others 2020), that show activity-dependent reversible remodeling and angiogenesis in hypothalamic and posterior pituitary microvessels (Alonso and others 2005; Miyata and Hatton 2002; Figure 3b and 3c). Similar morphologic adaptations have been reported in both regions in response to physiologic stimuli that elicit increased Oxt release, such as lactation, parturition, hemorrhage, restraint stress, as well as central administration of Oxt itself (Blanco and others 1991; Hatton 1988; Miyata and others 1994; Theodosis and others 1986; Tweedle and Hatton 1987). In the posterior pituitary, neurovascular reconstruction is driven by shape conversion of pericytes and astrocytes, increasing vascular surface area and permeability and enhancing direct contact of Oxt axonal terminals with the outer basement membrane (Furube and others 2014; Nishikawa and others 2017). These changes are accompanied by increased Oxt release and heightened expression of angiogenic factors in Oxt axonal terminals, astrocytes, ECs, and pericytes (Furube and others 2014; Nishikawa and others 2017; Figure 3c). Such remodeling in the pituitary presumably serves a secretory function by increasing the surface area for Oxt diffusion into peripheral circulation (and perhaps into CSF) to meet the high demand for Oxt, whereas morphologic plasticity in the hypothalamus is thought to have several functional consequences. For example, retraction of astrocytic processes can promote contact between Oxt neurons (facilitating paracrine somatodendritic release and diffusion), increase neuronal excitability via reductions in glutamate clearance and membrane potential, and concurrently allow synaptic remodeling and formation of new synapses onto Oxt neurons (Baudon and others 2022; Theodosis 2002; Theodosis and others 2006). Furthermore, increased blood delivery following angiogenesis could enhance the monitoring of osmotic agents such as sodium (Roy and others 2021), and elevated oxygen (O_2_) levels could serve to modulate neural activity (Zhang and others 2023).

Interestingly, inverse neurovascular coupling was discovered to induce positive feedback excitation of Avp neurons such that hyperosmotic stimulation (i.e., salt loading) progressively increased Avp release while evoking vasoconstriction that reduced local blood flow (Roy and others 2021). These seemingly contradictory neurovascular responses are compatible with the complementary actions of the two neuropeptides. While Oxt and Avp converge in their response to hyperosmolality (Balment and others 1986), they have opposite effects in plasma volume regulation: Oxt promotes volume contraction by modulating autonomic nervous system activity (Higa and others 2002) and inducing atrial natriuretic peptide release (Favaretto and others 1997), whereas Avp increases fluid retention and water intake (Kimura and others 1976; Szczepanska-Sadowska and others 1982). As neurosecretory cells synthesizing Oxt and Avp reside in the same hypothalamic nuclei, their local milieu must be under very stringent control to ensure the proper response to changes in osmolality and volume from both cell groups.

In support of this notion, it was previously shown that Avp and NO may be involved in the balance between vasoconstriction and vasodilation in response to osmotic stimulation (Du and others 2015). Blockade of V1aRs showing inverse neurovascular coupling unmasked underlying salt-induced vasodilation (Roy and others 2021) that could presumably be driven by Oxt neurons, which, upon osmotic stimulation, upregulate expression of neuronal NO synthase (Kadowaki and others 1994; Srisawat and others 2004), a modulator of Oxt and Avp MCN activity in various contexts, such as LTP maintenance and acute stress response (Matsuura and others 2023; Orlando and others 2007). In turn, basal Oxt and Avp release is tonically suppressed by NO-mediated potentiation of GABAergic inputs (Stern and Ludwig 2000). Conversely, under circumstances that elicit activity-dependent release, the inhibitory effect of NO on Avp is alleviated, while its suppression of Oxt secretion is acutely intensified (Antunes-Rodrigues and others 2004; Kadekaro and Summy-Long 2000). However, elevated O_2_ levels, which promote NO degradation (Zhang and others 2023), potentially boost Oxt MCN activity by alleviating NO-mediated negative feedback. Besides NO availability, O_2_ concentration directly regulates potassium channel expression and the rate of neuromodulator synthesis (Zhang and others 2023). Specifically, potassium channels TASK-1 and TASK-3 (Turner and Buckler 2013) as well as Kv3 channels (Kaczmarek and Zhang 2017) can be inhibited by declining O_2_ levels, leading to membrane depolarization and increased neuronal excitability. The presence of these potassium channels has been confirmed in hypothalamic MCNs (Han and others 2003; Kaczmarek and Zhang 2017), with stronger expression of some in Avp neurons (Shevchenko and others 2004). Therefore, increases in O_2_ concentrations may enhance Oxt activity, while declining O_2_ levels may promote Avp signaling, providing a basis for the antagonistic neurovascular coupling responses elicited by the two neuronal populations. Such distinct regulatory mechanisms likely fine-tune the hypothalamic microenvironment to establish a balance between Oxt and Avp and thus achieve stimulus-dependent neural discrimination.

### Blood Circulation and the BBB

Despite Oxt regulation of vasculature throughout the body (Gutkowska and Jankowski 2012; Gutkowska and others 2000), most research on Oxt and cerebrovasculature has focused on Oxt’s ability to permeate the BBB (Bowen 2019), as motivated by many studies and trials investigating the use of exogenous (specifically intranasal) Oxt for treatment of disorders such as autism spectrum disorder (Martins and others 2022). It was believed the BBB prevented the diffusion of Oxt in meaningful quantities from peripheral circulation into the brain and vice versa (Mens and others 1983), prompting mutually exclusive regulation of the two Oxt systems. Indeed, peripheral and central release of Oxt can occur independently (Landgraf and Neumann 2004); for example, dendritic release is thought to lag behind increases in plasma Oxt levels during osmotic stimulation (Neumann and others 2008). However, the cardiovascular system, namely the heart and large vessels, is able to synthesize Oxt in relatively large quantities (Gutkowska and others 1997; Jankowski and others 1998; Thibonnier and others 1999; Figure 1) and may contribute to the early rise in plasma levels prior to release in the pituitary, although this remains to be studied. Additionally, numerous physiologic cues, such as parturition, lactation, sexual activity, and stressors, can trigger Oxt to be released centrally and peripherally (Althammer and others 2021; Neumann and Landgraf 2012). Elicited behavioral effects, such as maternal care, anxiolysis, and social recognition, complement peripheral Oxt actions (i.e., labor and milk let-down; Engelmann and others 2004; Landgraf and others 1988; Neumann 2007). Nonetheless, co-release into plasma and CSF by collaterals of MCNs projecting to the pituitary can partially explain this phenomenon (Zhang and others 2021), and many Oxt-mediated behavioral effects (Jurek and Neumann 2018; Marsh and others 2021; Rigney and others 2022) are not accompanied by peripheral secretion (Althammer and others 2021).

Recent studies and meta-analyses point to a positive, albeit moderate and highly heterogeneous, correlation between Oxt concentrations in the CNS and periphery (Valstad and others 2017). While basal levels show no association, a positive correlation was observed after intranasal Oxt (IN-Oxt) treatment or introduction of experimental stressors, such as maternal separation (Kojima and others 2012) and forced swim test (Williams and others 2012), and was taken as supporting the hypothesis that basal Oxt levels in the blood and CNS are uncoordinated (Valstad and others 2017). The emergence of a correlation after experimental stressors could be attributed to hypothalamic-pituitary-adrenal axis activation, prompting interaction between corticotropin-releasing hormone PCNs and Oxt MCNs in the PVH (Ferguson and others 2008; Williams and others 2012) as well as autonomic feedback on the Oxt system via peripheral corticosteroid action (Quintana, Alvares, and others 2015). However, there are many caveats to such premature conclusions, as many issues have been reported with regard to the accurate measurement of peripheral plasma Oxt concentrations—specifically, discrepancies between radioimmunoassay and enzyme immunoassay methods that also depend on sample extraction (Szeto and others 2011; for extensive reviews and recommendations for best practice, see Leng and Sabatier 2016; MacLean and others 2019; D. Martins and others 2020; McCullough and others 2013; Tabak and others 2023). Furthermore, basal Oxt dynamics show oscillations that lead to tonic release (Ino and others 2022), and the half-life of Oxt is much shorter in blood (~3-8 minutes; Morin and others 2008) than in CSF (~20-30 minutes; Jones and Robinson 2008; Mens and others 1983). Therefore, even if peripheral and central release occurs simultaneously, random sampling of CSF and plasma could show discrepant concentrations due to differences in the timeline of Oxt degradation in the two media (also highlighted by Tabak and others 2023). In this line of reasoning, correlations detected after IN-Oxt administration or experimental stressors could be explained by stimulus-evoked release that is not constrained by baseline release dynamics. Moreover, sampling of CSF and plasma after experimental procedures would not be random but rather likely fall in the time window preceding degradation of plasma Oxt, leading to the detection of a correlation. Future research using sensors for real-time detection of Oxt release dynamics (Ino and others 2022; Qian and others 2023) and validated methods, such as combined two-dimensional liquid chromatography and mass spectrometry, to measure peripheral Oxt concentrations (Zhang and others 2011) could settle this debate on the correlation between peripheral and central Oxt (MacLean and others 2019; Tabak and others 2023). Moreover, establishing whether peripherally synthesized Oxt contributes to plasma Oxt levels and, if so, quantifying the extent of this contribution will aid in distinguishing increases attributable to release from the posterior pituitary (Assinder 2022; Figure 1).

**Figure 4.**
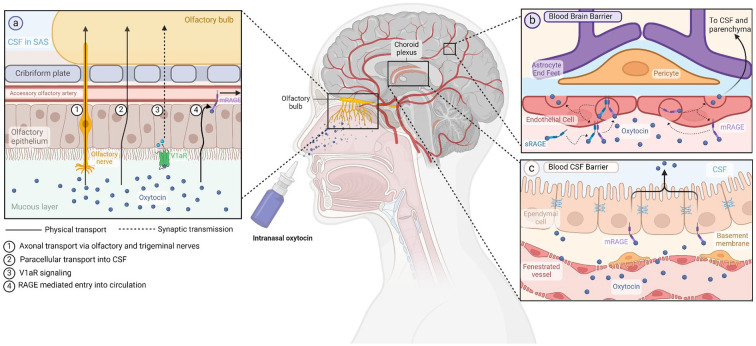
Oxytocin and proposed pathways across the blood-brain and blood-cerebrospinal fluid barriers. (a) Potential mechanisms of oxytocin absorption into the CNS following intranasal administration can involve direct delivery through axonal and paracellular transport, as well as indirect routes such as signal transduction following V1aR binding and RAGE-mediated uptake into nasal vessels. (b) In RAGE-mediated transport across the blood-brain barrier, oxytocin can either bind to membrane-bound RAGE on the basal side of vascular endothelial cells or attach to soluble RAGE, which then dimerizes with the membrane-bound form. The oxytocin-RAGE complex undergoes endocytosis into the cell and subsequent exocytosis to the apical side, facilitating diffusion into the neural parenchyma. (c) Oxytocin transport from plasma to CSF follows a similar process. At the blood-CSF barrier, oxytocin diffuses freely out of fenestrated vessels and is then shuttled from the basal to apical side of choroid plexus ependymal cells via RAGE. mRAGE = membrane-bound RAGE; RAGE = receptor for advanced glycation end products; SAS = subarachnoid space; sRAGE = soluble RAGE; V1aR = vasopressin receptor 1A. Created with BioRender.com.

Discrepant release dynamics and concentrations do not necessarily preclude an interaction between peripheral and central Oxt, especially in the setting of exogenous Oxt administration. Mode of delivery appears to be a key determinant, as indicated by findings of studies comparing IN-Oxt with intravenous (IV) Oxt administration, suggesting that while these methods of delivery lead to comparable peripheral Oxt concentrations, only IN-Oxt is able to elicit social cognitive effects (Quintana, Westlye, and others 2015) and neural effects (Quintana and others 2016), which implies IN-Oxt specific penetration of brain tissue. However, others reported that IV Oxt and IN-Oxt can activate Fos expression at the PVH, the area postrema, and the dorsal motor nucleus of the vagus (Maejima and others 2015). Importantly, the area postrema, located outside the BBB, is not accessible to CSF-Oxt, indicating a potential role for CVOs (Jeong and others 2021) for the detection of peripheral Oxt levels, as detailed later in this section. Similarly, increased Oxt levels in systemic circulation were found to explain Oxt-induced decreases in blood flow in the left amygdala and anterior cingulate cortex following IN-Oxt and IV Oxt (D. A. Martins and others 2020). Yet, this same study found additional changes in cerebral perfusion induced by IN-Oxt only, which could not be accounted for by changes in plasma Oxt, corroborating results from another group reporting that Oxt in amygdalar extracellular fluid was increased more by IN-Oxt delivery as compared with intraperitoneal (Smith and others 2019). In contrast, a recent clever experiment by Yao and colleagues (2023) demonstrated that the use of a vasoconstrictor pretreatment restricting IN-Oxt entry into systemic circulation not only impeded the increases in peripheral Oxt concentrations but also abolished a majority of resting-state neural responses to IN-Oxt, as measured by delta-beta cross-frequency coupling. These findings collectively suggest that peripheral routes of Oxt delivery are sufficient to elicit most central effects, while IN-Oxt may target additional brain regions, presumably via direct CSF penetration (Figure 4).

Strikingly, even with the delivery of very high concentrations of IN-Oxt, only negligible increases have been detected in CSF despite significant elevations in plasma levels (Born and others 2002; Chang and others 2012; Monte and others 2014). Based on these findings, two potential pathways of IN-Oxt entry into the brain were described, the first being axonal transport following uptake into olfactory and trigeminal neurons (Chen and others 1998; Thorne and others 1995, pathway 1) and the second consisting of passage into subarachnoid space (Born and others 2002; Illum 2000; Figure 4a). The first pathway could explain reports of low CSF levels following IN-Oxt delivery: Oxt is rapidly degraded in neural parenchyma, and only a fraction of centrally released Oxt reaches CSF (Born and others 2002; Thorne and others 1995); thus, it is possible that IN-Oxt could reach some brain regions via axonal transport without entering CSF. The second pathway suggests that IN-Oxt diffuses passively through perineural spaces of the nasal epithelium, bypassing the BBB entirely (Ermisch and others 1985; Fehm and others 2000; Illum 2000; Vyas and others 2005; Figure 4a, pathway 2). The finding that diverse patterns of Oxt-induced changes are seen in cerebral blood flow upon application of the same dose of IN-Oxt via standard nasal spray and nebulizer endorses this idea, as such differences can be related to small spatial variations in the deposition of IN-Oxt in paranasal cavities that make up entry points to the brain (D. A. Martins and others 2020).

However, the potential mechanisms of IN-Oxt are not limited to these two pathways. As mentioned, experimental use of extra physiologically high Oxt concentrations can lead to atypical effects via Oxt action at V1aRs (Chini and others 2017; Manning and others 2012; Neumann and Landgraf 2012). These receptors are expressed in the olfactory epithelium (Levasseur and others 2004), where Oxt binding could trigger signaling cascades back to the brain through synaptic pathways (Figure 4a, pathway 3, dashed line). Another explanation is that small but functionally significant amounts of Oxt can cross the BBB (Neumann and Landgraf 2012), exerting effects exclusively in areas with high Oxtr expression (e.g., amygdala; D. A. Martins and others 2020). Support for this hypothesis can be found in studies showing increased synthetic Oxt in CSF of primates after IV infusion of labeled synthetic Oxt despite no effects on endogenous release (Lee and others 2018). Similarly, intraperitoneal delivery of synthetic Oxt in wild type and Oxt knockout (KO) mice led to increases in extracellular fluid Oxt in the amygdala and dorsal hippocampus (Smith and others 2019). This hypothesis has gained further traction in the last few years with the discovery that the receptor for advanced glycation end products (RAGE) can act as an Oxt-specific transporter (Higashida and others 2017; Yamamoto and others 2019; Figure 4). RAGE was initially demonstrated to shuttle Oxt across the intestinal lumen, leading to absorption of Oxt from breastmilk into infant blood via intestinal permeability (Higashida and others 2017). This finding prompted studies of binding and transport characteristics of RAGE at the BBB, leading to the discovery that RAGE is expressed by vascular ECs and choroid plexus epithelium in the brain (Figure 4b and 4c) and mediates Oxt transport from peripheral circulation into the brain about 10 times more efficiently than the opposite direction (Figure 4a, pathway 4; Gerasimenko and others 2021; Leerach and others 2021; Munesue and others 2021; Shimizu and others 2020). Furthermore, this directional transport into the brain was obstructed in RAGE KO and knockdown mice, as demonstrated by little or no increase in CSF Oxt after IN-Oxt application (Yamamoto and others 2019). Notably, RAGE KO mice showed hyperactivity and deficits in maternal bonding (Yamamoto and others 2019) that were exacerbated by additional stress (Gerasimenko and others 2021), recapitulating behaviors seen in Oxt and Oxtr KO models as well as mice with deficiencies in Oxt secretion (i.e., CD38 KO; Ferguson and others 2000; Jin and others 2007; Takayanagi and others 2005). Importantly, these behavioral effects were not caused by a disruption of central Oxt signaling, as hypothalamic Oxt release was not affected by RAGE deficiency (Yamamoto and others 2019). Moreover, defects in Oxt transport and abnormalities in maternal behavior were rescued in RAGE KO mice upon transgenic EC expression of RAGE (Yamamoto and others 2019).

These findings suggest not only that peripheral Oxt can be transported into the brain but also that the delivered amounts are functionally meaningful, as the obstruction of such transport can abolish the central effects of exogenous Oxt (Yao and others 2023) and cause behavioral consequences without disruption of endogenous central Oxt signaling (Gerasimenko and others 2021; Yamamoto and others 2019). Munesue and colleagues (2021) proposed that transport of Oxt from peripheral circulation into the brain could be an alternative to volume transmission and en passant release in supplying areas without direct projections from Oxt neurons. The plausibility of this assertion is controversial, as the amount of Oxt that would need to be transported from the periphery into neural parenchyma to elicit behavioral and cognitive responses may be much higher than the transport capacity of RAGE alone, especially considering that Oxt cannot compete with the interaction of RAGE with other ligands (Leerach and others 2021). Moreover, despite Higashida and colleagues’ (2017) assertion that RAGE is expressed only by vascular ECs and choroid plexus epithelium in the brain, others have detected RAGE in a subset of neurons in the cerebral cortex (Brett and others 1993), with further studies showing RAGE involvement in embryonic and adult neuronal differentiation, neurite outgrowth, and nerve regeneration (Rong and others 2004; Wang and others 2008), as well as pathogenesis of neurologic disorders (Juranek and others 2022). It is therefore not yet possible to establish a causal relationship between the RAGE KO behavioral phenotype and impaired Oxt transport, even in EC-specific RAGE KO models, as RAGE is also implicated in vascular development and homeostasis (Adamopoulos and others 2016; Liu and others 2018) and disruption of RAGE signaling in cerebrovasculature would presumably affect neural function. That being said, RAGE is not the only candidate for an endogenous Oxt transporter: recent investigations by Værøy and colleagues (2023) indicate that a significant amount of plasma Oxt is naturally and reversibly bound to immunoglobulin G (IgG), a molecule capable of crossing the BBB (Chang and others 2018), and that the Oxt/IgG immune complexes are functionally active at the Oxtr, as demonstrated by calcium mobilization and receptor internalization assays (Værøy and others 2023). Furthermore, the same study showed that violent inmates have higher levels of Oxt-reactive IgG that are less efficient at carrying Oxt, as demonstrated by affinity kinetic parameters, which were correlated with psychological measures of aggressiveness. Overall, recent studies investigating potential transport mechanisms provide compelling support for the notion that peripheral Oxt reaches the brain and is essential for the complete spectrum of Oxt-induced behavioral and neural effects, although the exact amount of Oxt transported from blood to brain, as well as the details of such candidate mechanisms, remains to be fully elucidated. As with volume transmission, methods such as click chemistry or biosensors, which are minimally invasive and have superior resolution versus alternatives such as radiolabeling, fluorescence-based tagging, and microdialysis, could be utilized to determine whether peripherally administered Oxt reaches the brain (Ino and others 2022; Nakamura and others 2022; Qian and others 2023). Moreover, there are further avenues of interaction between neural tissue and peripheral circulation that are unrestricted by the BBB, which could supplement the proposed RAGE-mediated Oxt transport mechanism in various ways.

### Circumventricular Organs

**Figure 5.**
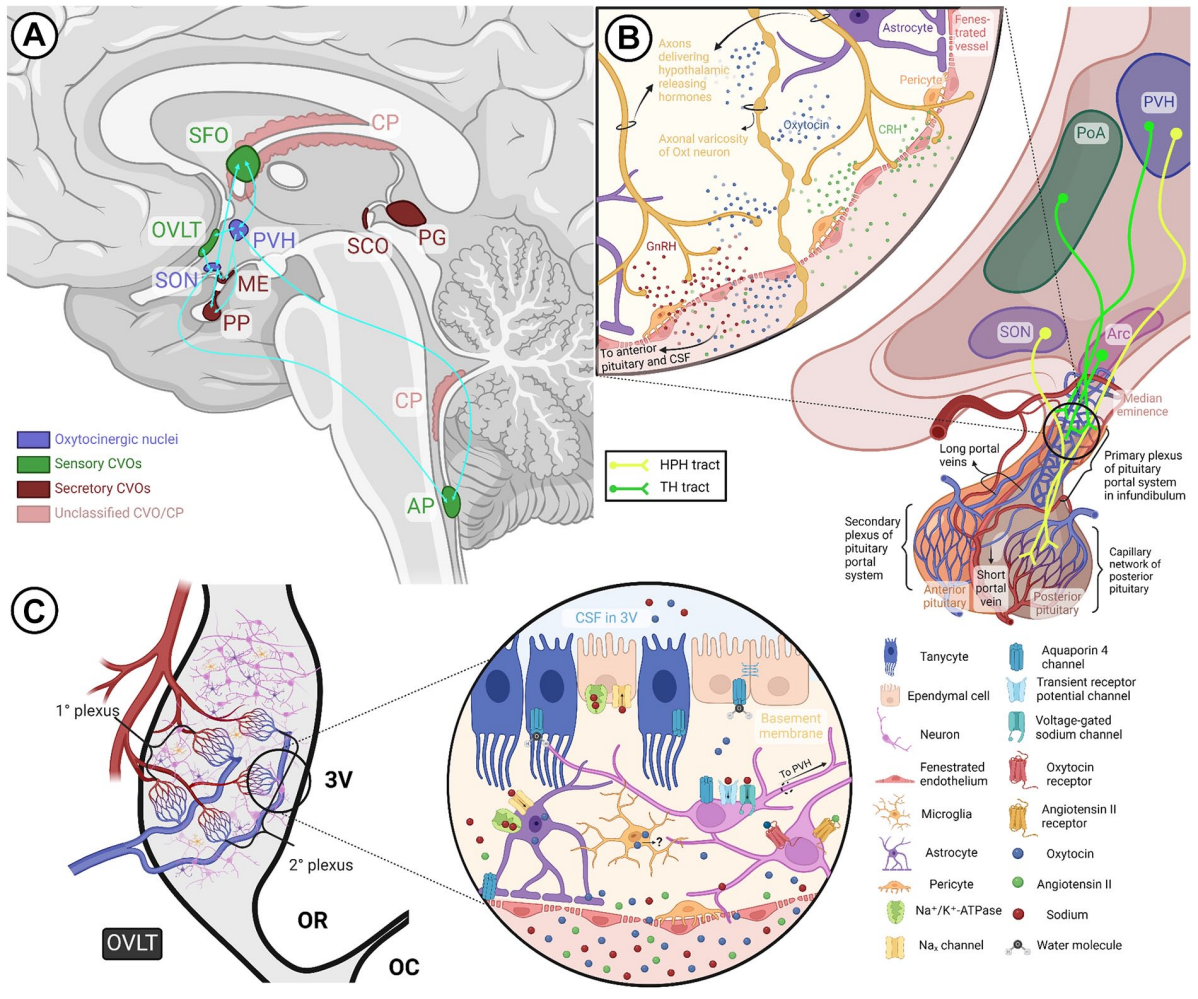
Circumventricular organs and the oxytocin system. (a) Hypothalamic oxytocinergic nuclei exhibit extensive reciprocal connections with select sensory CVOs, such as the SFO, OVLT, and AP, as well as certain secretory CVOs, such as the ME and PP. (b) The HPH tract and the hypophyseal portal system. The HPH tract containing oxytocinergic fibers courses through the median eminence and infundibulum before terminating as Herring bodies at the posterior pituitary capillary network. The primary plexus of the pituitary portal system, situated within the infundibular stalk, receives hypothalamic releasing hormones (e.g., GnRH, CRH) from the TH tract and delivers them to the anterior pituitary via the secondary plexus. As depicted in the callout, fenestrated vessels allow interaction of the two tracts at the level of the median eminence, primary plexus, and short portal vessels connecting the anterior and posterior pituitary. (c) Gross and magnified structure of the OVLT in relation to oxytocin signaling. Through its primary capillary networks, the OVLT makes contact with the subarachnoid space and third ventricle, where fenestrated vessels and a leaky blood-brain barrier allow monitoring of CSF and plasma osmolality via sodium channels, osmosensors, and other receptors present on neurons and glia. Additionally, some cells in the OVLT express oxytocin receptors and may even synthesize oxytocin. The OVLT and SFO communicate information regarding body fluid homeostasis to oxytocinergic nuclei, eliciting volume contraction and natriuresis via oxytocin signaling. 3V = third ventricle; AP = area postrema; Arc = arcuate nucleus; CP = choroid plexus; CRH = corticotropin-releasing hormone; CVO = circumventricular organ; GnRH = gonadotropin-releasing hormone; HPH = hypothalamoposthypophyseal; ME = median eminence; OC = optic chiasm; OR = optic recess; OVLT = organum vasculosum of lamina terminalis; Oxt = oxytocin; PG = pineal gland; PoA = preoptic area; PP = posterior pituitary; PVH = paraventricular nucleus of hypothalamus; SCO = subcommisural organ; SFO = subfornical organ; SON = supraoptic nucleus; TH = tuberohypophyseal. Created with BioRender.com.

CVOs are brain structures found in association with the third and fourth ventricles (Hofer 1958), distinguished by their fenestrated or otherwise permeable vascular network and proximity to the ventricular system (Petrov and others 1994; Wislocki and King 1936), making them a triadic interface among peripheral blood, CSF, and brain parenchyma (Sisó and others 2010). The proximity of all CVOs to either ventricular recesses or junctions and the orientation (and sometimes contact) of CVO-associated vessels and capillary loops toward ventricular cavities suggest a functional component to this physical relationship that can facilitate molecular interactions (Duvernoy and Risold 2007). There are 8 CVOs, although this number is contested (Duvernoy and Risold 2007; Oldfield and Mckinley 2004). Three of these—namely, the subfornical organ (SFO), the organum vasculosum of the lamina terminalis (OVLT), and the area postrema are sensory—while the neurohypophysis (or posterior pituitary), the median eminence, the pineal gland, and the subcommisural organ are considered secretory (Sisó and others 2010). The choroid plexus, which does not house any neural tissue, is sometimes referred to as a CVO, while the CVO identity of the subcommissural organ is questionable due to its lack of fenestrated endothelium (Petrov and others 1994). Sensory CVOs sample molecules from blood and interstitial fluid, relaying this information via projections to central hubs controlling autonomic functions, body fluid balance, and immune surveillance (Sisó and others 2010), implying significant overlap of CVO function with Oxt activity. Of note, all three sensory CVOs are in very close proximity to and/or have functional connectivity with hypothalamic nuclei that house Oxt neurons (Jeong and others 2021; Figure 5a).

OVLT, which is the sensory CVO closest to the SON and PVH, is positioned at the anteroventral border of the optic chiasm and serves as the vascular component of the lamina terminalis, constituting the anterior boundary of the 3V (Sisó and others 2010). Of the two primary capillary networks of OVLT, the superficial intrapial plexus associates with the subarachnoid space, while the deep plexus is located subependymally and connects to the 3V (Figure 5c; Shaver and others 1992). Ependymocytes and tanycytes, distinct types of specialized glial cells that line the ventricles of the brain (Duvernoy and Risold 2007), make up the ependymal lining at points of contact between the OVLT and ventricular recesses (Sisó and others 2010), earning CVOs the nickname “hypendymal organs” owing to these specialized glial networks, a lack of BBB, and high aquaporin 4 expression (Figure 5c; Goren and others 2006). While ependymocytes are linked by tight junctions (Duvernoy and Risold 2007), tanycytes extend processes that contact the CSF and the OVLT (Mullier and others 2010), presumably allowing the OVLT to track CSF composition. Functionally, the dorsal cap of the OVLT serves as a primary hub for efferent projections to various brain regions, including the SON, PVH, cerebral cortex, limbic structures, midbrain areas, and medullary raphe nucleus (Camacho and Phillips 1981; McKinley and others 1992). Highly relevant to Oxt and Avp actions in regulating blood volume and osmolality via natriuresis (Antunes-Rodrigues and others 2004), sodium-sensitive OVLT neurons monosynaptically communicate with the PVN (Shi and others 2008), and polysynaptic efferent pathways connecting the OVLT, PVH, and kidneys have been confirmed by experiments using viral tracers (McKinley and others 2003). Similarly, cells within the SFO interact with MCNs of the PVN directly as well as indirectly through projections to other hypothalamic nuclei, including the bed nucleus of the stria terminalis, arcuate nucleus, OVLT, and median preoptic nucleus (Abbott and others 2016; Kolaj and Renaud 2010; Matsuda and others 2017; Yeo and others 2019), with reciprocal connections between SFO and median preoptic nucleus potentially creating a feedback loop (Kawano 2017). Electrical stimulation of SFO is sufficient to induce Oxt and Avp release (Ferguson and Kasting 1987). In this vein, water deprivation, angiotensin I and II signaling, as well as hypertonic saline infusion and hyponatremia, which are all triggers for Oxt and Avp release, have been reported to activate OVLT and SFO osmoreceptors (Anderson and others 2000; Negoro and others 1988; Richard and Bourque 1995). Furthermore, OVLT and SFO neurons express high levels of angiotensin II type 1 receptors and angiotensin-converting enzyme (Giles and others 1999). Oxt-immunoreactive fibers have been observed in the OVLT, which were mostly traced back to astrocytes and basement membranes of the primary capillary network (Sisó and others 2010). Similarly, the superficial capillary plexus of the SFO shows Oxt staining, with immunohistochemical methods and mRNA measurements indicating that this signal might be coming from microglia (Blackmore and others 2018; Maejima and others 2022).

Such findings provide not only support for the notion that CVOs are functionally upstream of PVN and SON in establishing a balance of neuroendocrine-mediated metabolic pathways (Jeong and others 2021) but also raise questions regarding the purpose of Oxt expression in CVO glial cells of some species. Despite its classification as a sensory CVO, OVLT has been proposed to serve as a “double organ,” which acts as a sensor of molecules circulating in blood and CSF, as well as a secretory structure receiving dense afferent inputs from the hypothalamus (Oldfield and Mckinley 2004). This raises the unlikely but exciting probability that glial cells may not only sense but also secrete Oxt in CVOs (Maejima and others 2022). The purpose of such Oxt activity is up to debate; however, as with neurovascular reconstruction, it is possible that it may serve to modulate neuronal excitability. Oxt activity is known to increase BBB integrity by preventing excessive leakiness of the barrier (Momenabadi and others 2020; Stary and others 2019), and Oxt neurons themselves are able to sense the osmolarity of their environment, which increases their excitability (Mecawi and others 2022). Moreover, glial cells in the SFO were shown to regulate neuronal activity by sensing sodium levels in body fluids (Shimizu and others 2007). Oxt detection and secretion in CVOs could thus serve as a feedback mechanism aimed at regulating dispersion of peripheral signals in the CNS by 1) modulating afferent inputs from CVOs into hypothalamic nuclei and 2) monitoring the amount of osmolytes diffusing into CSF from circulation. Such regulatory mechanisms would serve to prevent hyperexcitability of Oxt neurons while ensuring optimal Oxt secretion in response to osmotic stimulation. The discovery of Oxtr expression in the OVLT, SFO, area postrema, and median eminence (Yoshida and others 2009) as well as the MPOA surrounding the OVLT (Sharma and others 2019) supports the notion that local Oxt signaling may serve to fine-tune afferent outputs from CVOs, potentially detecting and responding to peripheral Oxt that can freely diffuse into CVOs that lack a BBB.

Projections of Oxt MCNs are found within two of the secretory CVOs—namely, the structurally and functionally related neurohypophysis and the median eminence (Carey 1959; Currie and others 1960), implicating a role for these structures in Oxt release. The neurohypophysis (or posterior pituitary), originating from the floor of the 3V, consists of a distal part, the neural lobe, and a proximal part: the median eminence, separated by the hypophyseal recess (Duvernoy and Risold 2007). It is vascularized by an extensive capillary network receiving Oxt and Avp through the HPH tract (Figure 5b, yellow projections). The median eminence comprises an internal layer containing the HPH tract and an external layer receiving tuberohypophyseal fibers carrying releasing hormones from the hypothalamus to the adenohypophysis (or anterior pituitary; Yin and Gore 2010; Figure 5b, green projections), whereby the hypophyseal portal system facilitates one-way traffic of hormones, with a primary plexus receiving neurohormones and portal vessels reaching a secondary plexus vascularizing the adenohypophysis (Amar and Weiss 2003). The secondary plexus comprises long capillary loops that cross the external and internal layers of the median eminence. The fenestrated endothelium of these median eminence capillaries, coupled with large perivascular spaces (Wittkowski 1969), could allow interaction of the HPH tract with the tuberohypophyseal tract within these long capillaries (Jirikowski 2019), as well as within short portal vessels that link the lobes of the pituitary (Baertschi and others 1980; Bergland and Page 1978). Oxt and Avp are secretagogues of ACTH, which is released by the anterior pituitary upon corticotropin-releasing hormone signaling from the hypothalamus (Engelmann and others 2004), although how these neuropeptides reach the corticotrophs of the anterior pituitary is undetermined. It is likely that en passant release of Oxt and Avp in the median eminence (Buma and Nieuwenhuys 1987) could trigger ACTH secretion (Engelmann and others 2004; Figure 5b). Oxt and prolactin, another hormone of the anterior pituitary, are both involved in lactation and regulate the release of each other, with Oxt stimulating prolactin release (Egli and others 2006) and coordinating its pulsatile secretion via negative feedback (Bertram and others 2010), leading to the suggestion that Oxt might be the evasive prolactin-releasing hormone (Villegas-Gabutti and others 2018). If this is true, Oxt could enter the tuberohypophyseal system at the level of the median eminence and promote prolactin release in the anterior pituitary. Other than facilitating communication between the two hypothalamohypophyseal tracts, the long capillary loops of the secondary plexus reach the hypophyseal recess, suggesting a potential interaction of portal blood of the two tracts and CSF (Duvernoy and Risold 2007). The fenestrated capillaries of the median eminence contain tanycytes, which line the floor of the 3V and form a permeable layer, thus controlling barrier properties and allowing the exchange of molecules between CSF and parenchyma at the level of the arcuate nucleus (Mullier and others 2010). Axonal release at the level of the median eminence and perhaps posterior pituitary could therefore contribute to CSF Oxt levels and synchronize peripheral and central Oxt actions.

## Conclusion

Diverse yet interrelated mechanisms intricately regulate the transport and action of Oxt within the CNS. The coordinated interplay between axonal and somatodendritic Oxt release orchestrates rapid and localized physiologic, behavioral, and cognitive responses, complemented by volume transmission, which propagates Oxt signaling across the entire brain over an extended time frame. Here, we have highlighted the function of perivascular spaces as conduits for Oxt exchange between the CSF and neural parenchyma, exemplifying dynamic structural and functional adaptations induced by stimulus-driven increases in Oxt activity. While much emphasis has been placed on Oxt’s neuromodulatory role, its significant cardiovascular functions within the CNS have been comparatively overlooked. Exploring reciprocal interactions between the oxytocinergic and vascular systems in the brain holds promise for elucidating neurodevelopmental pathophysiology and age-related disorders, where concurrent alterations in both systems have been observed (El Assar and others 2012; Ghazy and others 2023; Ouellette and others 2020). Further investigations into these interactions hold promise for deepening our understanding of CNS function and introducing novel avenues for Oxt-based therapeutic interventions.

## Supplemental Material

sj-docx-1-nro-10.1177_10738584241268754 – Supplemental material for Navigating Central Oxytocin Transport: Known Realms and Uncharted Territories Supplemental material, sj-docx-1-nro-10.1177_10738584241268754 for Navigating Central Oxytocin Transport: Known Realms and Uncharted Territories by Deniz Parmaksiz and Yongsoo Kim in The Neuroscientist
