# Supplementary material for: Navigating Central Oxytocin Transport: Known Realms and Uncharted Territories
Source: Neuroscientist. 2024 Aug 7;31(3):234–61. doi: 10.1177/10738584241268754 (PMC12103645; doi:10.1177/10738584241268754)
Supplement: sj-docx-1-nro-10.1177_10738584241268754 – Supplemental material for Navigating Central Oxytocin Transport: Known Realms and Uncharted Territories [file sj-docx-1-nro-10.1177_10738584241268754.docx]

**Supplementary Table S1. Oxytocin and Oxytocin Receptor Expression in the Body.**

| **Site** | **References** |
| --- | --- |
| **Adipose Tissue** | **Boland and Goren, 1987; Yi and others, 2015; E. Li and others, 2024** |
| **Adrenal Glands** | **Ravid and others, 1986; Hawthorne and others, 1987; Cheliadinova and others, 2023.** |
| **Airway Smooth Muscle** | **Costa and others, 2006; Amrani and others, 2010.** |
| Bone | Copland and others, 1999; Tamma and others, 2009; Colaianni and others, 2011. |
| **Breast** | **Sapino and others, 1998; Cassoni and others, 2006.** |
| **Gravid uterus and placenta** | **Fields and others, 1983; Lefebvre and others, 1992; Chibbar and others, 1993; 1995; Yulia and Johnson, 2014, S.C. Kim and others, 2017.** |
| **Heart and vessels** | **Jankowski and others, 1998; 2000; Thibonnier and others, 1999; Gutkowska and others, 2000.** |
| **Liver** | **Kusui and others, 2001; Harricharran and Ogunwobi, 2019.** |
| **Kidneys** | **Stoeckel and others, 1987; Ostrowski and others, 1995; Shirley and others, 2011.** |
| **Muscle** | **Breton and others, 2002; De Jager and others, 2011; Divari and others, 2013; Berio and others, 2017.** |
| **Ovaries** | **Wathes and others, 1983; Furuya and others, 1995; Saller and others, 2010.** |
| **Pancreas** | **Amico and others, 1988; Suzuki and others, 2013; Mohan and others, 2018; Harricharran and Ogunwobi, 2020.** |
| **Prostate gland, epididymis, testis** | **Einspanier and Ivell, 1997; Assinder and others, 2000; 2004; Whittington and others, 2004; Gould and Nicholson, 2019.** |
| **Retina** | **Gauquelin and others, 1983; Halbach and others, 2015; Hu and others, 2022.** |
| **Skin** | **Denda and others, 2012; Deing and others, 2013; Fujimoto and others, 2023.** |
| **Spleen** | **Elands and others, 1990; Kumamoto and others, 1995.** |
| **Stomach and intestines** | **Monstein and others, 2004; Ohlsson and others, 2006; Qin and others, 2009; Welch and others, 2009; 2014; Danhof and others, 2023; Alqudah and others, 2023.** |
| **Thymus** | **Geenen and others, 1986; Hansenne and others, 2005.** |

Additional References for Supplementary Table S1:

Alqudah, M., Razzaq, R. A., Alfaqih, M. A., Al-Shboul, O., Al-Dwairi, A., & Taha, S. (2022). Mechanism of Oxytocin-Induced Contraction in Rat Gastric Circular Smooth Muscle. International journal of molecular sciences, 24(1), 441. https://doi.org/10.3390/ijms24010441

Amico, J. A., Finn, F. M., & Haldar, J. (1988). Oxytocin and vasopressin are present in human and rat pancreas. The American journal of the medical sciences, 296(5), 303–307. https://doi.org/10.1097/00000441-198811000-00003

Amrani Y, Syed F, Huang C, Li K, Liu V, Jain D, Keslacy S, Sims MW, Baidouri H, Cooper PR, Zhao H, Siddiqui S, Brightling CE, Griswold D, Li L, Panettieri RA Jr. Expression and activation of the oxytocin receptor in airway smooth muscle cells: Regulation by TNFalpha and IL-13. Respir Res. 2010 Jul 29;11(1):104. doi: 10.1186/1465-9921-11-104.

Assinder, S. J., Carey, M., Parkinson, T., & Nicholson, H. D. (2000). Oxytocin and vasopressin expression in the ovine testis and epididymis: changes with the onset of spermatogenesis. Biology of reproduction, 63(2), 448–456. https://doi.org/10.1095/biolreprod63.2.448

Assinder, S. J., Johnson, C., King, K., & Nicholson, H. D. (2004). Regulation of 5alpha-reductase isoforms by oxytocin in the rat ventral prostate. Endocrinology, 145(12), 5767–5773. https://doi.org/10.1210/en.2004-0711

Berio, E., Divari, S., Starvaggi Cucuzza, L., Biolatti, B., & Cannizzo, F. T. (2017). 17β-estradiol upregulates oxytocin and the oxytocin receptor in C2C12 myotubes. PeerJ, 5, e3124. https://doi.org/10.7717/peerj.3124

Boland, D., & Goren, H. J. (1987). Binding and structural properties of oxytocin receptors in isolated rat epididymal adipocytes. Regulatory peptides, 18(1), 7–18. <https://doi.org/10.1016/0167-0115(87)90045-0>

[Breton, C., Haenggeli, C., Barberis, C., Heitz, F., Bader, C. R., Bernheim, L., & Tribollet, E. (2002). Presence of functional oxytocin receptors in cultured human myoblasts. The Journal of clinical endocrinology and metabolism, 87(3), 1415–1418. https://doi.org/10.1210/jcem.87.3.8537](https://doi.org/10.1016/0167-0115(87)90045-0)

[Cassoni, P., Marrocco, T., Sapino, A., Allia, E., & Bussolati, G. (2006). Oxytocin synthesis within the normal and neoplastic breast: first evidence of a local peptide source. International journal of oncology, 28(5), 1263–1268.](https://doi.org/10.1016/0167-0115(87)90045-0)

[Cheliadinova, U., Pevnev, G., Ryu, V., Frolinger, T., Lee Sims, S., Ofer, M., Korkmaz, F., Barak, O., Gimenez Roig, J., Sultana, F., Kramskiy, N., Wizman, S., Orloff, M., Yuen, T., Lizneva, D., Zaidi, M., & Gumerova, A. (2023). OR27-05 Two-pronged Mediation Of Adrenal Steroidogenesis By Oxytocin. Journal of the Endocrine Society, 7(Suppl 1), bvad114.1768.](https://doi.org/10.1016/0167-0115(87)90045-0)

<https://doi.org/10.1210/jendso/bvad114.1768>

Chibbar, R., Miller, F. D., & Mitchell, B. F. (1993). Synthesis of oxytocin in amnion, chorion, and decidua may influence the timing of human parturition. The Journal of clinical investigation, 91(1), 185–192. https://doi.org/10.1172/JCI116169

Chibbar, R., Wong, S., Miller, F. D., & Mitchell, B. F. (1995). Estrogen stimulates oxytocin gene expression in human chorio-decidua. The Journal of clinical endocrinology and metabolism, 80(2), 567–572. https://doi.org/10.1210/jcem.80.2.7852522

Colaianni, G., Sun, L., Di Benedetto, A., Tamma, R., Zhu, L. L., Cao, J., Grano, M., Yuen, T., Colucci, S., Cuscito, C., Mancini, L., Li, J., Nishimori, K., Bab, I., Lee, H. J., Iqbal, J., Young, W. S., 3rd, Rosen, C., Zallone, A., & Zaidi, M. (2012). Bone marrow oxytocin mediates the anabolic action of estrogen on the skeleton. The Journal of biological chemistry, 287(34), 29159–29167. https://doi.org/10.1074/jbc.M112.365049

Copland, J. A., Ives, K. L., Simmons, D. J., & Soloff, M. S. (1999). Functional oxytocin receptors discovered in human osteoblasts. Endocrinology, 140(9), 4371–4374. https://doi.org/10.1210/endo.140.9.7130

[Costa, E. L., Schettino, I. A., & Schettino, G. P. (2006). The lung in sepsis: guilty or innocent?. Endocrine, metabolic & immune disorders drug targets, 6(2), 213–216. https://doi.org/10.2174/187153006777442413](https://doi.org/10.1016/0167-0115(87)90045-0)

[Danhof, H. A., Lee, J., Thapa, A., Britton, R. A., & Di Rienzi, S. C. (2023). Microbial stimulation of oxytocin release from the intestinal epithelium via secretin signaling. Gut microbes, 15(2), 2256043. https://doi.org/10.1080/19490976.2023.2256043](https://doi.org/10.1016/0167-0115(87)90045-0)

[De Jager, N., Hudson, N. J., Reverter, A., Wang, Y. H., Nagaraj, S. H., Cafe, L. M., Greenwood, P. L., Barnard, R. T., Kongsuwan, K. P., & Dalrymple, B. P. (2011). Chronic exposure to anabolic steroids induces the muscle expression of oxytocin and a more than fiftyfold increase in circulating oxytocin in cattle. Physiological genomics, 43(9), 467–478. https://doi.org/10.1152/physiolgenomics.00226.2010](https://doi.org/10.1016/0167-0115(87)90045-0)

[Denda, S., Takei, K., Kumamoto, J., Goto, M., Tsutsumi, M., & Denda, M. (2012). Oxytocin is expressed in epidermal keratinocytes and released upon stimulation with adenosine 5'-[γ-thio]triphosphate in vitro. Experimental dermatology, 21(7), 535–537.](https://doi.org/10.1016/0167-0115(87)90045-0)

<https://doi.org/10.1111/j.1600-0625.2012.01507.x>

[Deing, V., Roggenkamp, D., Kühnl, J., Gruschka, A., Stäb, F., Wenck, H., Bürkle, A., & Neufang, G. (2013). Oxytocin modulates proliferation and stress responses of human skin cells: implications for atopic dermatitis. Experimental dermatology, 22(6), 399–405. https://doi.org/10.1111/exd.12155](https://doi.org/10.1016/0167-0115(87)90045-0)

[Divari, S., Pregel, P., Cannizzo, F. T., Starvaggi Cucuzza, L., Brina, N., & Biolatti, B. (2013). Oxytocin precursor gene expression in bovine skeletal muscle is regulated by 17β-oestradiol and dexamethasone. Food chemistry, 141(4), 4358–4366. https://doi.org/10.1016/j.foodchem.2013.07.029](https://doi.org/10.1016/0167-0115(87)90045-0)

[Einspanier, A., & Ivell, R. (1997). Oxytocin and oxytocin receptor expression in reproductive tissues of the male marmoset monkey. Biology of reproduction, 56(2), 416–422. https://doi.org/10.1095/biolreprod56.2.416](https://doi.org/10.1016/0167-0115(87)90045-0)

[Elands, J., Resink, A., & De Kloet, E. R. (1990). Neurohypophyseal hormone receptors in the rat thymus, spleen, and lymphocytes. Endocrinology, 126(5), 2703–2710. https://doi.org/10.1210/endo-126-5-2703](https://doi.org/10.1016/0167-0115(87)90045-0)

[Fields, P. A., Eldridge, R. K., Fuchs, A. R., Roberts, R. F., & Fields, M. J. (1983). Human placental and bovine corpora luteal oxytocin. Endocrinology, 112(4), 1544–1546.](https://doi.org/10.1016/0167-0115(87)90045-0)

<https://doi.org/10.1210/endo-112-4-1544>

[Fujimoto, K., Inada, K., Oka, K., & Ito, E. (2023). Revisiting oxytocin generation in keratinocytes. Biophysics and physicobiology, 20(1), e200003. https://doi.org/10.2142/biophysico.bppb-v20.0003](https://doi.org/10.1016/0167-0115(87)90045-0)

[Furuya, K., Mizumoto, Y., Makimura, N., Mitsui, C., Murakami, M., Tokuoka, S., Ishikawa, N., Imaizumi, E., Katayama, E., & Seki, K. (1995). Gene expressions of oxytocin and oxytocin receptor in cumulus cells of human ovary. Hormone research, 44 Suppl 2, 47–49. https://doi.org/10.1159/000184661](https://doi.org/10.1016/0167-0115(87)90045-0)

[Gauquelin, G., Geelen, G., Louis, F., Allevard, A. M., Meunier, C., Cuisinaud, G., Benjanet, S., Seidah, N. G., Chretien, M., & Legros, J. J. (1983). Presence of vasopressin, oxytocin and neurophysin in the retina of mammals, effect of light and darkness, comparison with the neuropeptide content of the neurohypophysis and the pineal gland. Peptides, 4(4), 509–515. https://doi.org/10.1016/0196-9781(83)90056-6](https://doi.org/10.1016/0167-0115(87)90045-0)

[Geenen, V., Legros, J. J., Franchimont, P., Baudrihaye, M., Defresne, M. P., & Boniver, J. (1986). The neuroendocrine thymus: coexistence of oxytocin and neurophysin in the human thymus. Science (New York, N.Y.), 232(4749), 508–511. https://doi.org/10.1126/science.3961493](https://doi.org/10.1016/0167-0115(87)90045-0)

[Gould, M. L., & Nicholson, H. D. (2019). Changes in receptor location affect the ability of oxytocin to stimulate proliferative growth in prostate epithelial cells. Reproduction, fertility, and development, 31(6), 1166–1179. https://doi.org/10.1071/RD18362](https://doi.org/10.1016/0167-0115(87)90045-0)

[Halbach P, Pillers DA, York N, Asuma MP, Chiu MA, Luo W, Tokarz S, Bird IM, Pattnaik BR. Oxytocin expression and function in the posterior retina: a novel signaling pathway. Invest Ophthalmol Vis Sci. 2015 Jan 15;56(2):751-60. doi: 10.1167/iovs.14-15646.](https://doi.org/10.1016/0167-0115(87)90045-0)

[Hansenne, I., Rasier, G., Péqueux, C., Brilot, F., Renard, C.h, Breton, C., Greimers, R., Legros, J. J., Geenen, V., & Martens, H. J. (2005). Ontogenesis and functional aspects of oxytocin and vasopressin gene expression in the thymus network. Journal of neuroimmunology, 158(1-2), 67–75. https://doi.org/10.1016/j.jneuroim.2004.08.007](https://doi.org/10.1016/0167-0115(87)90045-0)

[Harricharran, T., & Ogunwobi, O. O. (2019). Oxytocin receptor genetic alterations in hepatocellular carcinoma. SN comprehensive clinical medicine, 1(7), 523–526. https://doi.org/10.1007/s42399-019-00085-2](https://doi.org/10.1016/0167-0115(87)90045-0)

[Harricharran, T., & Ogunwobi, O. O. (2020). Oxytocin and oxytocin receptor alterations, decreased survival, and increased chemoresistance in patients with pancreatic cancer. Hepatobiliary & pancreatic diseases international : HBPD INT, 19(2), 175–180. https://doi.org/10.1016/j.hbpd.2019.12.002](https://doi.org/10.1016/0167-0115(87)90045-0)

[Hawthorn, J., Nussey, S. S., Henderson, J. R., & Jenkins, J. S. (1987). Immunohistochemical localization of oxytocin and vasopressin in the adrenal glands of rat, cow, hamster and guinea pig. Cell and tissue research, 250(1), 1–6. https://doi.org/10.1007/BF00214646](https://doi.org/10.1016/0167-0115(87)90045-0)

[Hu S, Wang Y, Han X, Dai M, Zhang Y, Ma Y, Weng S, Xiao L. Activation of oxytocin receptors in mouse GABAergic amacrine cells modulates retinal dopaminergic signaling. BMC Biol. 2022 Sep 21;20(1):205. doi: 10.1186/s12915-022-01405-0.](https://doi.org/10.1016/0167-0115(87)90045-0)

[Jankowski, M., Wang, D., Hajjar, F., Mukaddam-Daher, S., McCann, S. M., & Gutkowska, J. (2000). Oxytocin and its receptors are synthesized in the rat vasculature. Proceedings of the National Academy of Sciences of the United States of America, 97(11), 6207–6211. https://doi.org/10.1073/pnas.110137497](https://doi.org/10.1016/0167-0115(87)90045-0)

[Kim, S. C., Lee, J. E., Kang, S. S., Yang, H. S., Kim, S. S., & An, B. S. (2017). The regulation of oxytocin and oxytocin receptor in human placenta according to gestational age. Journal of molecular endocrinology, 59(3), 235–243. https://doi.org/10.1530/JME-16-0223](https://doi.org/10.1016/0167-0115(87)90045-0)

[Kumamoto, K., Matsuura, T., Amagai, T., & Kawata, M. (1995). Oxytocin-producing and vasopressin-producing eosinophils in the mouse spleen: immunohistochemical, immuno-electron-microscopic and in situ hybridization studies. Cell and tissue research, 281(1), 1–10. https://doi.org/10.1007/BF00307953](https://doi.org/10.1016/0167-0115(87)90045-0)

[Kusui, C., Kimura, T., Ogita, K., Nakamura, H., Matsumura, Y., Koyama, M., Azuma, C., & Murata, Y. (2001). DNA methylation of the human oxytocin receptor gene promoter regulates tissue-specific gene suppression. Biochemical and biophysical research communications, 289(3), 681–686. https://doi.org/10.1006/bbrc.2001.6024](https://doi.org/10.1016/0167-0115(87)90045-0)

[Lefebvre, D. L., Giaid, A., Bennett, H., Larivière, R., & Zingg, H. H. (1992). Oxytocin gene expression in rat uterus. Science (New York, N.Y.), 256(5063), 1553–1555. https://doi.org/10.1126/science.1598587](https://doi.org/10.1016/0167-0115(87)90045-0)

Li, E., Wang, L., Wang, D., Chi, J., Lin, Z., Smith, G. I., Klein, S., Cohen, P., & Rosen, E. D. (2024). Control of lipolysis by a population of oxytocinergic sympathetic neurons. Nature, 625(7993), 175–180. <https://doi.org/10.1038/s41586-023-06830-x>

Mohan, S., Khan, D., Moffett, R. C., Irwin, N., & Flatt, P. R. (2018). Oxytocin is present in islets and plays a role in beta-cell function and survival. Peptides, 100, 260–268. https://doi.org/10.1016/j.peptides.2017.12.019

Monstein, H. J., Grahn, N., Truedsson, M., & Ohlsson, B. (2004). Oxytocin and oxytocin-receptor mRNA expression in the human gastrointestinal tract: a polymerase chain reaction study. Regulatory peptides, 119(1-2), 39–44. https://doi.org/10.1016/j.regpep.2003.12.017

Ohlsson, B., Truedsson, M., Djerf, P., & Sundler, F. (2006). Oxytocin is expressed throughout the human gastrointestinal tract. Regulatory peptides, 135(1-2), 7–11. https://doi.org/10.1016/j.regpep.2006.03.008

Ostrowski, N. L., Young, W. S., 3rd, & Lolait, S. J. (1995). Estrogen increases renal oxytocin receptor gene expression. Endocrinology, 136(4), 1801–1804. https://doi.org/10.1210/endo.136.4.7895693

Qin, J., Feng, M., Wang, C., Ye, Y., Wang, P. S., & Liu, C. (2009). Oxytocin receptor expressed on the smooth muscle mediates the excitatory effect of oxytocin on gastric motility in rats. Neurogastroenterology and motility, 21(4), 430–438. https://doi.org/10.1111/j.1365-2982.2009.01282.x

Ravid R, Oosterbaan HR Hansen BL, Swaab DF (1986) Localization of oxytocin, vasopressin and part of precursors in the human neonatal adrenal. Histochemistry 84:401-407. <https://doi.org/10.1007/BF00482970>

Saller, S., Kunz, L., Dissen, G. A., Stouffer, R., Ojeda, S. R., Berg, D., Berg, U., & Mayerhofer, A. (2010). Oxytocin receptors in the primate ovary: molecular identity and link to apoptosis in human granulosa cells. Human reproduction (Oxford, England), 25(4), 969–976. https://doi.org/10.1093/humrep/dep467

Sapino, A., Cassoni, P., Stella, A., & Bussolati, G. (1998). Oxytocin receptor within the breast: biological function and distribution. Anticancer research, 18(3C), 2181–2186.

Shirley, D. G., Walter, M. F., Keeler, B. D., Waters, N. J., & Walter, S. J. (2011). Selective blockade of oxytocin and vasopressin V(1a) receptors in anaesthetised rats: evidence that activation of oxytocin receptors rather than V(1a) receptors increases sodium excretion. Nephron. Physiology, 117(3), p21–p26. https://doi.org/10.1159/000320290

Stoeckel, M. E., Freund-Mercier, M. J., Palacios, J. M., Richard, P., & Porte, A. (1987). Autoradiographic localization of binding sites for oxytocin and vasopressin in the rat kidney. The Journal of endocrinology, 113(2), 179–182. https://doi.org/10.1677/joe.0.1130179

Suzuki, M., Honda, Y., Li, M. Z., Masuko, S., & Murata, Y. (2013). The localization of oxytocin receptors in the islets of Langerhans in the rat pancreas. Regulatory peptides, 183, 42–45. https://doi.org/10.1016/j.regpep.2013.03.019

Tamma, R., Colaianni, G., Zhu, L. L., DiBenedetto, A., Greco, G., Montemurro, G., Patano, N., Strippoli, M., Vergari, R., Mancini, L., Colucci, S., Grano, M., Faccio, R., Liu, X., Li, J., Usmani, S., Bachar, M., Bab, I., Nishimori, K., Young, L. J., … Zallone, A. (2009). Oxytocin is an anabolic bone hormone. Proceedings of the National Academy of Sciences of the United States of America, 106(17), 7149–7154. <https://doi.org/10.1073/pnas.0901890106>

[Wathes, D. C., Swann, R. W., Birkett, S. D., Porter, D. G., & Pickering, B. T. (1983). Characterization of oxytocin, vasopressin, and neurophysin from the bovine corpus luteum. Endocrinology, 113(2), 693–698. https://doi.org/10.1210/endo-113-2-693](https://doi.org/10.1016/0167-0115(87)90045-0)

[Welch, M. G., Tamir, H., Gross, K. J., Chen, J., Anwar, M., & Gershon, M. D. (2009). Expression and developmental regulation of oxytocin (OT) and oxytocin receptors (OTR) in the enteric nervous system (ENS) and intestinal epithelium. The Journal of comparative neurology, 512(2), 256–270. https://doi.org/10.1002/cne.21872](https://doi.org/10.1016/0167-0115(87)90045-0)

[Welch, M. G., Margolis, K. G., Li, Z., & Gershon, M. D. (2014). Oxytocin regulates gastrointestinal motility, inflammation, macromolecular permeability, and mucosal maintenance in mice. American journal of physiology. Gastrointestinal and liver physiology, 307(8), G848–G862. https://doi.org/10.1152/ajpgi.00176.2014](https://doi.org/10.1016/0167-0115(87)90045-0)

[Whittington, K., Assinder, S., Gould, M., & Nicholson, H. (2004). Oxytocin, oxytocin-associated neurophysin and the oxytocin receptor in the human prostate. Cell and tissue research, 318(2), 375–382. https://doi.org/10.1007/s00441-004-0968-5](https://doi.org/10.1016/0167-0115(87)90045-0)

Yi, K. J., So, K. H., Hata, Y., Suzuki, Y., Kato, D., Watanabe, K., Aso, H., Kasahara, Y., Nishimori, K., Chen, C., Katoh, K., & Roh, S. G. (2015). The regulation of oxytocin receptor gene expression during adipogenesis. Journal of neuroendocrinology, 27(5), 335–342. <https://doi.org/10.1111/jne.12268>

Yulia, A., & Johnson, M. R. (2014). Myometrial oxytocin receptor expression and intracellular pathways. Minerva ginecologica, 66(3), 267–280.
